# Consequences of temporary inhibition of the medial amygdala on social recognition memory performance in mice

**DOI:** 10.3389/fnins.2015.00152

**Published:** 2015-04-29

**Authors:** Julia Noack, Rita Murau, Mario Engelmann

**Affiliations:** Center for Behavioral Brain Sciences, Institut für Biochemie und Zellbiologie, Otto-von-Guericke-Universität MagdeburgMagdeburg, Germany

**Keywords:** male C57BL/6JOlaHsd mice, social long-term recognition memory, medial amygdala, olfaction, lesion, lidocaine, behavior, learning and memory

## Abstract

Different lines of investigation suggest that the medial amygdala is causally involved in the processing of information linked to social behavior in rodents. Here we investigated the consequences of temporary inhibition of the medial amygdala by bilateral injections of lidocaine on long-term social recognition memory as tested in the social discrimination task. Lidocaine or control NaCl solution was infused immediately before learning or before retrieval. Our data show that lidocaine infusion immediately before learning did not affect long-term memory retrieval. However, intra-amygdalar lidocaine infusions immediately before choice interfered with correct memory retrieval. Analysis of the aggressive behavior measured simultaneously during all sessions in the social recognition memory task support the impression that the lidocaine dosage used here was effective as it—at least partially—reduced the aggressive behavior shown by the experimental subjects toward the juveniles. Surprisingly, also infusions of NaCl solution blocked recognition memory at both injection time points. The results are interpreted in the context of the importance of the medial amygdala for the processing of non-volatile odors as a major contributor to the olfactory signature for social recognition memory.

## Introduction

Individual recognition in socially living rodents is primarily based on the acquisition and processing of the conspecifics' body odors. They are thought to contain information about age, sex, reproductive state and the health status and, thus, differ individually and they are often referred to as the “olfactory signature.” Physico-chemically, body odors are composed of volatile and non-volatile fractions. Once acquired by the individual, the neuronal processing of such “olfactory signatures” involves distinct brain areas linked to both the main and the accessory olfactory system. One of the most important brain areas involved in the processing of olfactory signals is the medial amygdala (MeA) which has been suggested to play an important role in olfactory social stimulus processing in rodents (Lehman et al., 1980; Ferguson et al., 2001; Broad et al., 2002; Curtis and Wang, 2003; Gobrogge et al., 2007; Gutierrez-Castellanos et al., 2014). Not only the analysis of c-Fos synthesis (Ferguson et al., 2001; Richter et al., 2005; Noack et al., 2010), but also genetic and pharmacological (Ferguson et al., 2001) studies revealed that this brain area may play a key role for social recognition in mice. In this context the MeA was shown to act not only as a relay station from the olfactory bulb to deeper brain areas, but also to signal back to the accessory olfactory bulb thereby controlling the impact of the non-volatile fraction of the conspecific's “olfactory signature” on approach-avoidance behavior (Martel and Baum, 2009). However, it is unclear what might be the consequences of temporarily blocking the information processing in the MeA during acquisition/consolidation of olfactory memory vs. its recall. Therefore, we investigated the effects of bilateral temporary suppression of action potential propagation in the MeA by injecting Lidocaine into the MeA prior to both learning and recall of the individual olfactory information.

Some of the results were previously published in abstract form (Noack et al., 2012).

## Materials and methods

### Experimental subjects

Male C57BL/6JOlaHsd mice (originally obtained from Harlan-Winkelmann, Bochern, Germany and subsequently bred in the animal facility of the Medical faculty of the Otto-von-Guericke-Universität Magdeburg), aged 9–16 weeks, were kept in groups of 5 under a constant 12 h:12 h light-dark cycle (light starting at 6 a.m.) with food and water available *ad libitum*.

Juvenile C57BL/6JOlaHsd mice of both sexes (age 25–35 days) were used as social stimuli. The suitability of these social stimuli for the behavioral test used was previously proven in intensive studies (Engelmann et al., 2011).

All procedures and manipulations were approved by the local governmental body and according to the European Communities Council Directive recommendations for the care and use of laboratory animals (2010/63/EU).

### Social discrimination procedure

**Figure 1:**
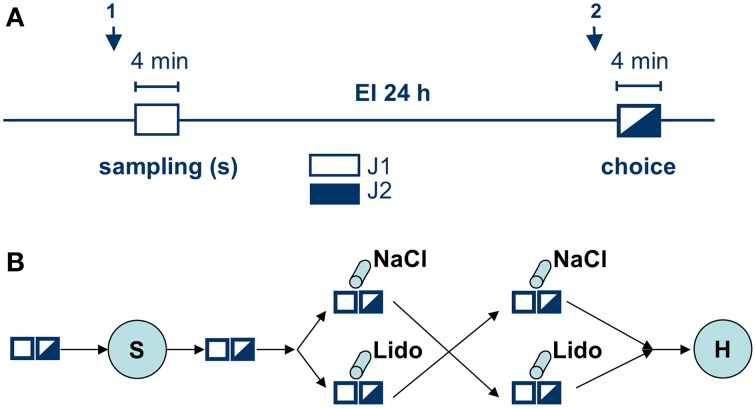
**Experimental protocol. (A)** shows the social discrimination procedure. Arrows indicate time points for intra-amygdalar injections either directly before sampling (1) or before choice (2); both sampling (white rectangle) and choice (black-white rectangle) lasted 4 min and were separated by a 24 h exposure interval (EI). During sampling a given Juvenile (J1, white) was exposed to the experimental subject. During choice J1 (encountered during sampling) together with a novel, previously not encountered juvenile (J2, black) were exposed to the experimental subject and the given behavioral parameters toward each juvenile measured. **(B)** shows the whole experimental series involving all manipulations and illustrating the cross-over design. White and black-white rectangles illustrate the two sessions in social discrimination, S, surgery to implant the guide cannulas, cylinders indicate intra-amygdalar injections of NaCl or lidocaine (Lido) during behavioral testing; H, histological analysis.

Experimental subjects were separated 2 h before testing. The test procedure consisted of two sessions (4 min each) separated by a 24 h exposition interval (Figure 1A). During the first session (sampling), a conspecific juvenile (J1) was presented to the experimental subject, allowing the adult to acquire the juvenile's “olfactory signature.” During the second session (choice) J1 was re-exposed to the adult together with a second, previously not encountered juvenile (J2). A significantly longer investigation duration of J2 vs. J1 during choice was taken as an evidence for an intact long-term social recognition memory (LTsrM) (Engelmann et al., 2011). In addition, sexual (e.g., attempts of mounting) and aggressive behavior [e.g., chasing and biting the juvenile(s)] were separately monitored (see Engelmann et al., 2011 for more details).

#### Surgery

Animals were briefly anesthetized with isoflurane (2%, Baxter, Unterschleiß heim, Germany, applied via an anesthesia system (MLW, Leipzig, Germany) by a constant flow of 1.2 l/min and then injected with a mixture of ketamine and xylazine (i.p., 2 ml Ketavet® (Pfizer Pharmacia, Berlin, Germany), 0.5 ml Rompun® (Bayer Vital, Leverkusen, Germany), 7.5 ml 0.9% NaCl solution). Deeply anesthetized animals were fixed into a stereotaxic frame (TSE Systems, Bad Homburg, Germany). Coordinates for bilateral implantation of the guide cannulas (stainless surgical steel 0.55 × 0.08 × 9.00 mm; Injecta, Klingenthal, Germany) were selected according to a stereotaxic mouse brain atlas (Franklin and Paxinos, 1997). The ventral tip of each guide cannula was placed 1 mm above the target area, the MeA, at the following coordinates starting from Bregma: lateral ± 2.5 mm, anterior 0.3 mm, ventral 4.5 mm. The guide cannulas were secured to the skull and to two stainless steel screws (1.0 × 2.0 mm, Paul Korth, Lüdenscheid, Germany) inserted into the bone with light curling dental cement (Ivoclar Vivadent, Schaan, Lichtenstein). Stylets were made from insect pins and inserted into the guide cannulas to keep them patent. Animals received an injection of Meloxicam (s.c., 0.05 mg/kg b. wt., Metacam® Boehringer Ingelheim Vetmedica, Ingelheim, Germany) to temper post-operative pain.

**Figure 2:**
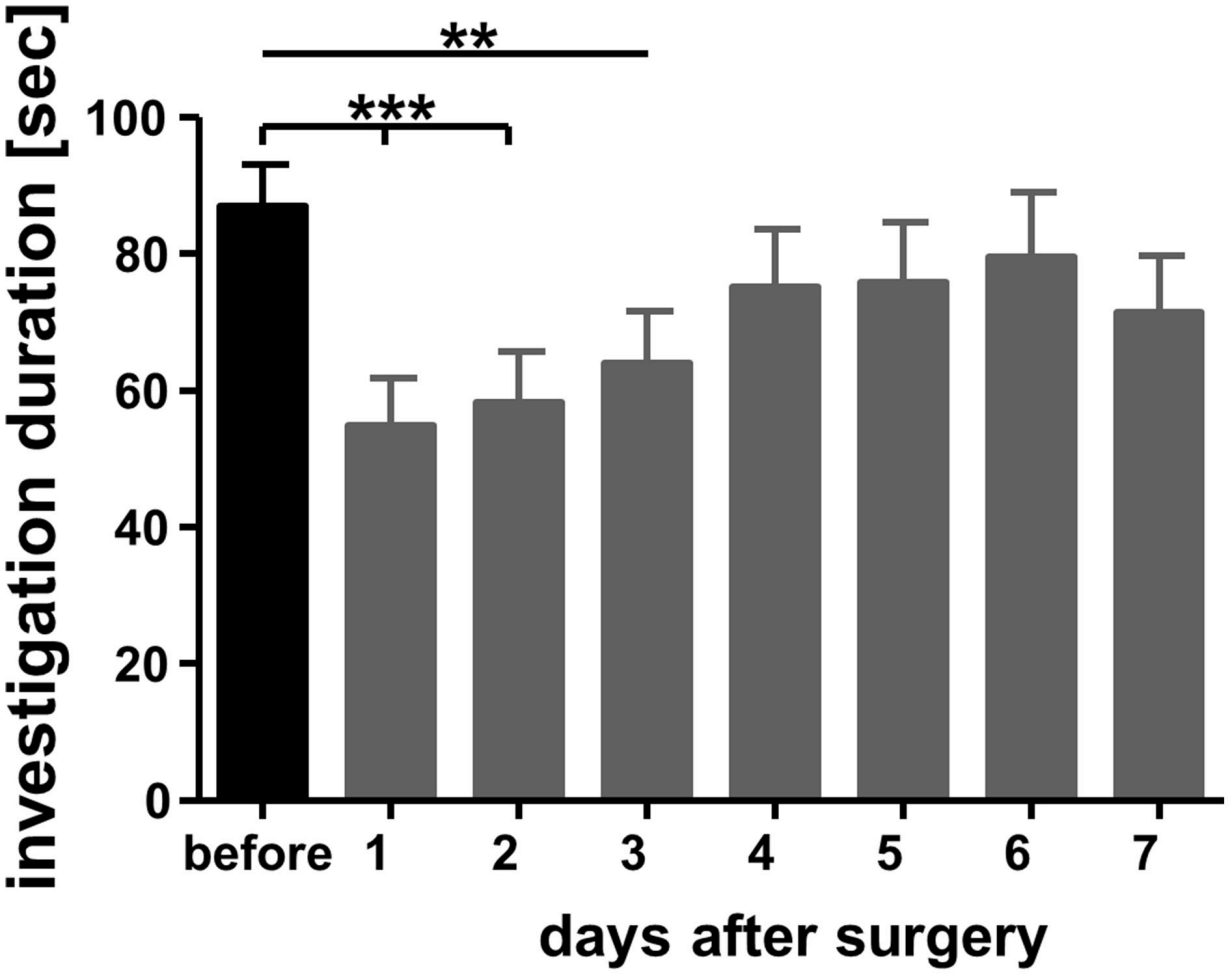
**Investigation durations measured a day before bilateral implantation of guide cannulas targeting the medial amygdala and on the subsequent days**. Adult male mice were exposed in their home cages for 4 min toward a previously not encountered conspecific juvenile similar to sampling in the social discrimination procedure (for more details see text). A trained observed measured the duration spent by the experimental subjects investigating the juveniles. ^**^p < 0.01 and ^***^p < 0.01 compared with the investigation duration measured during “before.” Repeated measures ANOVA followed by Dunnett's-test.

Experimental subjects were allowed to recover from surgery for at least 7 days before the experiment. Pilot studies revealed that at this time point both body weight and social behavior reached pre-surgery levels (Figure 2).

#### Acute injections, substances, and treatment

For simultaneous acute bilateral injections animals were removed from the cage, anesthetized with isoflurane (see above) and placed on a table. Stylets were removed and injection cannulas gently inserted into the guide cannulas. Substances were infused via a constant flow of 0.5 μl/min using a microinfusion pump (CMA Microdialysis, Stockholm, Sweden). The proper application was controlled by watching the movement of an airbubble within a scaled tubing connecting the microinfusion pump with the injection cannula. We infused a volume of 0.5 μl into each hemisphere. After the infusion injection cannulas were left in place for an additional minute to allow for complete delivery. Subsequently, anesthesia was stopped, the injection cannulas were carefully replaced by the stylets and the animals were returned to their experimental cages. Given the dosage of Lidocaine (Xylocitin-loc®, Mibe, Jena, Germany; 20 μg/μl diluted in 0.9% NaCl solution, Braun, Melsungen, Germany) used we calculated an inhibition of action potential generation and propagation by blocking voltage-gated sodium channels, duration of effect: <60 min (Malpeli and Schiller, 1979; Boehnke and Rasmusson, 2001).

### Experimental design

Before undergoing surgery experimental subjects used in this study were tested for their intact long-term social discrimination abilities. Seven days after surgery the animals were tested for their social discrimination abilities without any further treatment to confirm that the surgery and the implantation of the guide cannulas did not cause effects (e.g., by lesions) that *per se* interfered with a successful long-term recognition memory.

Recent reports suggest interfering effects of both isoflurane anesthesia (Pearce et al., 2012) and transport of animals between rooms (Moura et al., 2011) on olfactory memory performance. Therefore, we tested the impact of the manipulations linked to the acute intra-amygdalar injections on the behavioral parameters as measured in the present study. Our data revealed that the anesthesia procedure *per se* did not produce interfering effects for LTsrM if applied according to our experimental protocol (Noack, unpublished observations, Camats Perna, unpublished observations). This is in line with previous reports that the brief isoflurane-anesthesia performed at different time intervals before and after sampling fails to corrupt long-term juvenile recognition abilities in male mice of the mouse strain under study *per se* (Richter et al., 2005; Wanisch et al., 2008; Engelmann, 2009; Engelmann et al., 2011).

As shown in Figure 1B, infusions of lidocaine or NaCl were performed in a double-blind, cross-over approach either (1) directly before sampling or (2) directly before choice. The code for the substances was randomly assigned to each experiment separately by a co-worker not involved in the infusion and behavioral testing. The blinding was broken at the end of the histological verification of the placement of the injection side. Recovery time between infusion and the onset of the respective sampling and choice session respectively was ~ 6 min. The same group of animals (*n* = 15, divided into two subgroups according to the protocol shown in Figure 1B) received the infusion of lidocaine and NaCl (cross-over) before sampling. Another group of animals (*n* = 17, also divided into two subgroups and treated according to Figure 1B) received the infusions (cross-over) before choice. Thus, each animal in the respective group received both lidocaine and NaCl. The interval between the two successive treatments (NaCl followed by lidocaine or lidocaine followed by NaCl; Figure 1B) was 7 days.

#### Statistics

Data are presented as means + SEM. For the analysis of the aggressive/sexual behavior data were submitted in a non-parametric Friedmans ANOVA followed by Wilcoxon corrected for repeated measures. For the analysis of the investigation duration measured during sampling data were analyzed using paired student's *t*-tests. Statistics were performed using GraphPad Prism 4.0 (GraphPad Software Inc., La Jolla, U.S.A.). A *p* < 0.05 was considered to indicate statistical significance.

## Results

### Aggressive/sexual behavior

**Figure 3:**
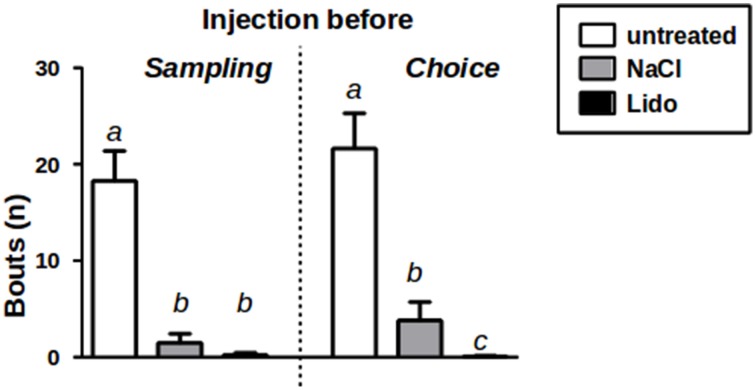
**Aggression/Sexual behavior toward J1 during sampling w/o injection (untreated) and after injection of NaCl or Lidocaine (Lido) directly before sampling (left panel, *n* = 15)**. Aggression/Sexual behavior toward J1+J2 during choice w/o injection and after injection of NaCl or Lido directly before choice (right panel, *n* = 17). a = *p* < 0.01 vs. b and c; c = *p* < 0.05 vs. b, Friedman-test followed by Wilcoxon corrected for repeated measures.

We observed that the durations spent in aggressive behavior were—at least under treatment conditions—too low to allow for a reliable analysis (average duration of an investigation bout <0.5 s). Therefore, we analyzed the number of bouts instead since it has been shown to provide a more reliable measure in cases of low durations of individual investigation bouts (Engelmann et al., 2011). Our data show that—compared to implanted, but otherwise untreated conditions—both NaCl and lidocaine infusions significantly reduced the number of investigation bouts of aggressive/sexual behavior (Friedmans ANOVA: *Fs* = 26.53, *p* < 0.01; Figure 3). Furthermore, under lidocaine treatment aggressive/sexual behavior decreased almost completely reaching statistical significance—compared to NaCl treatment—if the animals were injected immediately before choice (Friedmans ANOVA: *Fs* = 24.14, *p* < 0.01; Figure 3).

### Social discrimination

**Figure 4:**
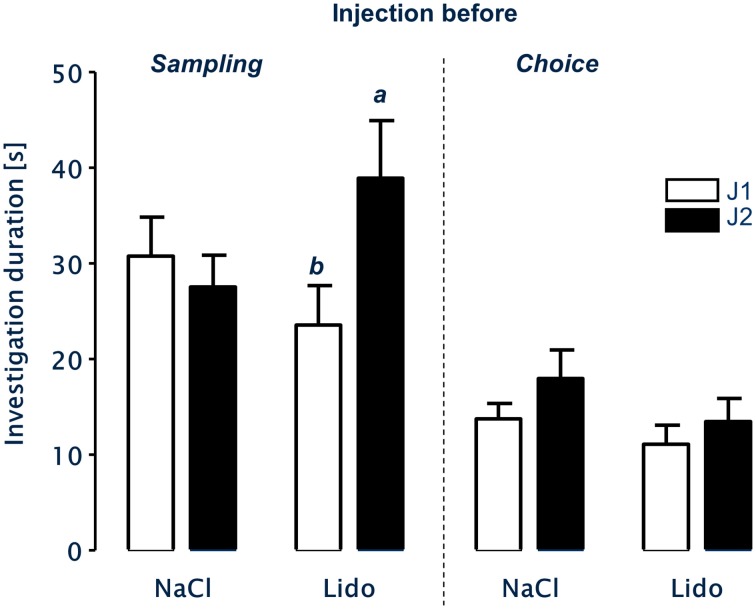
**LTsrM in mice after injection of NaCl or Lidocaine (Lido) immediately before either sampling (left panel, *n* = 15) or choice (right panel, *n* = 17)**. Mice treated with NaCl before sampling or choice failed to recognize the previously encountered juvenile. Injection of Lidocaine directly before sampling did not affect LTsrM. In contrast, injection of Lidocaine directly before choice impaired LTsrM. b = *p* < 0.05 vs. a, paired student's *t*-test.

After injection of NaCl-solution immediately before sampling there was no significant difference in the investigation durations of the experimental subjects toward both juveniles J1 and J2 during choice (paired *T*-test, *t* = 1.099, *df* = 14, *p* = 0.29; Figure 4). In contrast, the same animals investigated J2 significantly longer than J1 during choice after receiving the injection of lidocaine directly before sampling (paired *T*-test, *t* = 2.648, *df* = 14, *p* = 0.019).

After receiving injections of NaCl or lidocaine immediately before choice, the experimental subjects showed no significant differences in the duration of investigation behavior toward J1 and J2 during choice (NaCl: paired *T*-test, *t* = 1.270, *df* = 16, *p* = 0.222; lidocaine: paired *T*-test, *t* = 1.050, *df* = 16, *p* = 0.309; Figure 4).

## Discussion

The present study was designed to investigate the impact of temporary inhibition of the medial amygdala on LTsrM. Our data suggest that the combination of isoflurane anesthesia with direct bilateral infusions of different drugs is suitable to investigate the impact for example of temporary lesions of distinct brain areas for LTsrM in mice. More specifically, the protocol used here seems to avoid the interfering effects of isoflurane anesthesia reported by other authors (Pearce et al., 2012).

Previous studies have shown that permanent lesions of the medial amygdala significantly reduced aggressive behavior not only in rats (Vochteloo and Koolhaas, 1987), but also in C57Bl/6 mice (Wang et al., 2013). Aggressive behavior is also seen—albeit in comparably low intensity—during the social discrimination testing where 13–35% of the total social behavior was considered to be aggressive and of a sexual nature (Engelmann et al., 2011). The almost complete lack of aggressive/sexual behavior toward the stimulus juveniles after administration of lidocaine (see Figure 3) implies that lidocaine administration inhibited local neurosignaling that in turn resulted in a suppression of aggressive/sexual behavior. This observation is important with respect to the effects observed for recognition memory: Lidocaine administration failed to interfere with LTsrM if administered immediately before sampling and blocked the memory performance if administered immediately before choice (Figure 4). Thus, temporarily lesioning the medial amygdala during acquiring the olfactory signature does not affect juvenile recognition.

Interestingly, intra-amygdalar injection of the NaCl-solution alone (originally thought to act as “neutral” control) reduced aggressive behavior (Figure 3) and impaired LTsrM (Figure 4). This implies that the NaCl-solution triggered behavioral effects that tended to be similar to that of lidocaine. Indeed, there is some evidence that infusion of the NaCl-solution also lowers electrophysiological activity in a short-term manner (Malpeli and Schiller, 1979; Tehovnik and Sommer, 1997). Nevertheless, as shown in Figure 3 the behavioral suppressive effect of NaCl solution treatment was not as pronounced as that of lidocaine. This may have resulted not only in the reduced aggressive activity toward the juveniles (Figure 3), but also in an improper processing of the “olfactory signature” during sampling. This is in line with results of a study investigating among other contextual fear conditioning that revealed that injections of NaCl-solutions into the lateral amygdala mimicked partially effects of lidocaine when compared with sham injections (Calandreau et al., 2005). It may be speculative, but we propose that the resulting modified “olfactory signature” was artificial and could not match with the correctly processed “olfactory signature” obtained during the choice session, resulting in an inability to recognize the originally encountered juvenile, and thus the reported lack of an intact LTsrM.

Lidocaine should completely block electrical activity in the MeA, beyond any abovementioned effect of NaCl, and this is supported by the aggression/sexual behavioral data. We observed that lidocaine injection before sampling did not interfere with LTsrM. However, when injected immediately before choice successful recognition of the previously encountered juvenile was blocked (Figure 4). These observations were surprising and led us to the conclusion that information processing within the MeA can be blocked without interfering with the successful acquisition of olfactory information important for LTsrM (electrical block during sampling). According to our results however, the MeA is likely to act as an essential relay station for information during recall after being activated during learning (electrical block only during choice). In addition, an even more detailed interpretation of the different consequences of lidocaine treatment for LTsrM “before sampling” vs. “before choice” may relate to the fact that the MeA is thought to be primarily involved in the processing of non-volatile olfactory cues (for review see Baum and Bakker, 2013). Previously it was shown that the non-volatile fraction of the “olfactory signature” is not essential for a correct social memory retrieval, if not available during sampling, but it must be available for successful LTsrM during choice when it was accessible during sampling (Noack et al., 2010). Our present results seem to mirror these findings by blocking the information processing of the non-volatile fraction of the olfactory signature in the MeA. Blocking the transfer/processing of information in this brain area by lidocaine, but not NaCl, during learning does not affect LTsrM when both fractions are accessible during retrieval. In contrast, when both fractions of the olfactory signature were processed successfully during sampling (including the non-volatile fraction in the MeA) the blockade (lidocaine) and the modulation (NaCl) of the non-volatile odor processing in the MeA interfered with LTsrM. This hypothesis might be an attractive target to further investigate the phenomenon of interference in further studies.

Taken together, the data of the present study suggest that the MeA seems to play an—albeit dispensable—role in the processing of the non-volatile-fraction of the “olfactory signature” for LTsrM. Blocking the generation of action potentials in the MeA during retrieval also blocks LTsrM. Therefore, our data point toward a contribution of the MeA in the processing of complete “olfactory signatures” for social behavior. These observations illustrate the plasticity of the neuronal substrate for processing stimuli relevant for social recognition memory in order to compensate for the temporary lesion of an otherwise essential brain area.

## Author contributions

ME and JN planned the experiments and wrote the manuscript. JN and RM performed the experiments.

### Conflict of interest statement

The authors declare that the research was conducted in the absence of any commercial or financial relationships that could be construed as a potential conflict of interest.

## References

[B1] Baum M. J. Bakker J. (2013). Roles of sex and gonadal steroids in mammalian pheromonal communication. Front. Neuroendocrinol. 34:268–284. 10.1016/j.yfrne.2013.07.004 23872334

[B2] Boehnke S. E. Rasmusson D. D. (2001). Time course and effective spread of lidocaine and tetrodotoxin delivered via microdialysis: an electrophysiological study in cerebral cortex. J. Neurosci. Methods 105, 133–141. 10.1016/S0165-0270(00)00348-4 11275270

[B3] Broad K. D. Mimmack M. L. Keverne E. B. Kendrick K. M. (2002). Increased BDNF and trk-B mRNA expression in cortical and limbic regions following formation of a social recognition memory. Eur. J. Neurosci. 16, 2166–2174. 10.1046/j.1460-9568.2002.02311.x 12473084

[B4] Calandreau L. Desmedt A. Decorte L. Jaffard R. (2005). A different recruitment of the lateral and basolateral amygdala promotes contextual or elemental conditioned association in Pavlovian fear conditioning. Learn. Mem. 12, 383–388. 10.1101/lm.92305 16027178 PMC1183256

[B5] Curtis J. T. Wang Z. (2003). Forebrain c-fos expression under conditions conducive to pair bonding in female prairie voles (Microtus ochrogaster). Physiol. Behav. 80, 95–101. 10.1016/S0031-9384(03)00226-9 14568313

[B6] Engelmann. (2009). Competition between two memory traces for long-term recognition memory. Neurobiol. Learn. Mem. 91, 58–65. 10.1016/j.nlm.2008.08.009 18812226

[B7] Engelmann M. Hädicke J. Noack J. (2011). Testing declarative memory in laboratory rats and mice using the non-conditioned social discrimination procedure. Nat. Protoc. 6, 1152–1162. 10.1038/nprot.2011.353 21799485

[B8] Ferguson J. N. Aldag J. M. Insel T. R. Young L. J. (2001). Oxytocin in the medial amygdala is essential for social recognition in the mouse. J. Neurosci. 21, 8278–8285. 11588199 10.1523/JNEUROSCI.21-20-08278.2001 PMC6763861

[B9] Franklin K. B. J. Paxinos G. (1997). The Mouse Brain in Stereotaxic Coordinates. San Diego, CA: Academic Press.

[B10] Gobrogge K. L. Liu Y. Jia X. Wang Z. (2007). Anterior hypothalamic neural activation and neurochemical associations with aggression in pair-bonded male prairie voles. J. Comp. Neurol. 502, 1109–1122. 10.1002/cne.21364 17444499

[B11] Gutierrez-Castellanos N. Pardo-Bellver C. Martinez-Garcia F. Lanuza E. (2014). The vomeronasal cortex—afferent and efferent projections of the posteromedial cortical nucleus of the amygdala in mice. Eur. J. Neurosci. 39, 141–158. 10.1111/ejn.12393 24188795

[B12] Lehman M. N. Winans S. S. Powers J. B. (1980). Medial nucleus of the amygdala mediates chemosensory control of male hamster sexual behavior. Science 210, 557–560. 10.1126/science.7423209 7423209

[B13] Malpeli J. G. Schiller P. H. (1979). A method of reversible inactivation of small regions of brain tissue. J. Neurosci. Methods 1, 143–151. 10.1016/0165-0270(79)90011-6 120911

[B14] Martel K. L. Baum M. J. (2009). A centrifugal pathway to the mouse accessory olfactory bulb from the medial amygdala conveys gender-specific volatile pheromonal signals. Eur. J. Neurosci. 29, 368–376. 10.1111/j.1460-9568.2008.06564.x 19077123 PMC2754263

[B15] Moura P. J. Venkitaramani D. V. Tashev R. Lombroso P. J. Xavier G. F. (2011). Transport of animals between rooms: a little-noted aspect of laboratory procedure that may interfere with memory. Behav. Processes 88, 12–19. 10.1016/j.beproc.2011.06.008 21729741

[B16] Noack J. Murau R. Engelmann M. (2012). The medial amygdala in learning and recall of long-term social recognition memory in mice: a temporary lesion study, in FENS Forum Abstr 2012 (Barcelona).

[B17] Noack J. Richter K. Laube G. Haghgoo H. A. Veh R. W. Engelmann M. (2010). Different importance of the volatile and non-volatile fractions of an olfactory signature for individual social recognition in rats versus mice and short-term versus long-term memory. Neurobiol. Learn. Mem. 94, 568–575. 10.1016/j.nlm.2010.09.013 20888419

[B18] Pearce R. A. Duscher P. Van Dyke K. Lee M. Andrei A. C. Perouansky M. (2012). Isoflurane impairs odour discrimination learning in rats: differential effects on short- and long-term memory. Br. J. Anaesth. 108, 630–637. 10.1093/bja/aer451 22258200 PMC3303486

[B19] Richter K. Wolf G. Engelmann M. (2005). Social recognition memory requires two stages of protein synthesis in mice. Learn. Mem. 12, 407–413 10.1101/lm.97505 16077019 PMC1183259

[B20] Tehovnik E. J. Sommer M. A. (1997). Effective spread and timecourse of neural inactivation caused by lidocaine injection in monkey cerebral cortex. J. Neurosci. Methods 74, 17–26. 10.1016/S0165-0270(97)02229-2 9210571

[B21] Vochteloo J. D. Koolhaas J. M. (1987). Medial amygdala lesions in male rats reduce aggressive behavior: interference with experience. Physiol. Behav. 41, 99–102. 10.1016/0031-9384(87)90137-5 3685168

[B22] Wang Y. He Z. Zhao C. Li L. (2013). Medial amygdala lesions modify aggressive behavior and immediate early gene expression in oxytocin and vasopressin neurons during intermale exposure. Behav. Brain Res. 245, 42–49. 10.1016/j.bbr.2013.02.002 23403283

[B23] Wanisch K. Wotjak C. T. Engelmann M. (2008). Long-lasting second stage of recognition memory consolidation in mice. Behav. Brain Res. 186, 191–196. 10.1016/j.bbr.2007.08.008 17875328

